# Multi-trait multi-environment quantitative trait loci mapping for a sugarcane commercial cross provides insights on the inheritance of important traits

**DOI:** 10.1007/s11032-015-0366-6

**Published:** 2015-08-09

**Authors:** G. R. A. Margarido, M. M. Pastina, A. P. Souza, A. A. F. Garcia

**Affiliations:** Departamento de Genética, Escola Superior de Agricultura “Luiz de Queiroz” (ESALQ), Universidade de São Paulo (USP), CP 83, Piracicaba, SP 13418-900 Brazil; Embrapa Milho e Sorgo, CP 285, Sete Lagoas, MG 35701-970 Brazil; Centro de Biologia Molecular e Engenharia Genética (CBMEG), Departamento de Genética e Evolução, Universidade Estadual de Campinas (UNICAMP), Cidade Universitária Zeferino Vaz, CP6010, Campinas, SP 13083-875 Brazil

**Keywords:** Multiple interval mapping, Full-sib family, Genetic architecture, Model selection, Polyploid

## Abstract

Breeding trials typically consist of phenotypic observations for various traits evaluated in multiple environments. For sugarcane in particular, repeated measures are obtained for plant crop and one or more ratoons, such that joint analysis through mixed models for modeling heterogeneous genetic (co)variances between traits, locations and harvests is appropriate. This modeling approach also enables us to include molecular marker information, aiding in understanding the genetic architecture of quantitative traits. Our work aims at detecting QTL and QTL by environment interactions by fitting mixed models with multiple QTLs, with appropriate modeling of multi-trait multi-environment data for outcrossing species. We evaluated 100 individuals from a biparental cross at two locations and three  years for fiber content, sugar content (POL) and tonnes of cane per hectare (TCH). We detected 13 QTLs exhibiting QTL by location, QTL by harvest or the three-way interaction. Overall, 11 of the 13 effects presented some degree of pleiotropy, affecting at least two traits. Furthermore, these QTLs always affected fiber and TCH in the same direction, whereas POL was affected in the opposite way. There was no evidence in favor of the linked QTL over the pleiotropic QTL hypothesis for any detected genome position. These results provide valuable insights into the genetic basis of quantitative variation in sugarcane and the genetic relation between traits.

**Electronic supplementary material:**

The online version of this article (doi:10.1007/s11032-015-0366-6) contains supplementary material, which is available to authorized users.

## Introduction

Plant breeding is an essential activity to agriculture, affording short-term new elite cultivars and long-term potential for increased yields and response to adversities (Moose and Mumm 2008). To select both broadly stable genotypes and those adapted to specific environmental conditions, it is paramount to have information about genotype by environment interaction (G × E) and, particularly for marker-assisted selection (MAS), QTL × E interaction (Eeuwijk et al. 2001; Smith et al. 2001; Verbyla et al. 2003; Boer et al. 2007). Likewise, knowledge of the genetic correlations between traits can provide clues as to the possibility of breaking undesirable correlations between agronomically important traits, therefore playing an important role when designing breeding strategies (Jiang and Zeng 1995; Welham et al. 2010).

To achieve such goals, evaluation trials are generally conducted in several locations, ideally with contrasting environmental features, and throughout many (consecutive) years. For species that can be vegetatively propagated, such as sugarcane, repeated measures can be obtained for clones kept in the field, for both plant crop and ratoons (Smith et al. 2007). The ability to evaluate the same genotypes in multiple sites and years makes such data naturally suited for joint analysis. In particular, appropriate modeling of genetic and residual (co)variance matrices alleviates the need to make unrealistic assumptions about the distribution of errors and affords unbiased estimates of model effects, such as QTL effects (Balzarini 2001). In this context of modeling G × E interaction and correlation between traits, mixed models are a natural statistical approach to use (Smith et al. 2005).

It can be observed from the breeding field trial evaluation literature that few studies jointly analyze multi-trait multi-environment (MTME) data (Malosetti et al. 2008). The most likely reason for this is the difficulty in analyzing the data, particularly in interpreting the results, as well as the lack of adequate and easily accessible statistical methodology and software for such goals. Traditionally, data of this type have been analyzed by fitting separate ordinary or somehow naïve models for each trait and environment combination, multi-environment models for each trait or multi-trait models individually for each environment (Sun et al. 2012; Bonneau et al. 2013; Freeman et al. 2013; Lopes et al. 2013; Liu et al. 2014), followed by informal comparisons, generally graphical, or *ad hoc* meta-analysis to derive conclusions about G × E and/or correlation between traits (Piepho 2000). The situation is particularly evident for QTL studies: only a handful of examples utilize MTME modeling for QTL mapping, even for well-studied species for which inbred-derived populations are available (Malosetti et al. 2006, 2008; Singh et al. 2012; Alimi et al. 2013).

Furthermore, another important aspect is that most QTL mapping studies disregard the occurrence of epistasis. This phenomenon has occasionally been overlooked, considered rare and less important than other genetic effects, but collective evidence attests its prevalence and importance (Garcia et al. 2008; Phillips 2008; Manolio et al. 2009; Eichler et al. 2010). QTL mapping can benefit from the inclusion of epistatic interactions directly in the search process, such as that by the multiple interval mapping (MIM) model (Kao and Zeng 1997; Kao et al. 1999). This practice increases statistical power for QTL detection, removes biases from QTL effect and position estimates and yields breeding values that can be directly leveraged by breeding programs through MAS (Zeng et al. 1999; Collard and Mackill 2008). The MIM method can be interpreted as a model (variable) selection procedure and, as such, can be readily incorporated into the mixed model context through a least-squares approximation (Haley and Knott 1992; Broman and Sen 2009).

The situation for sugarcane is not unlike that encountered for most diploid species, albeit with a few extra obstacles. Most studies make use of two linkage maps, one for each parent, constructed based on markers segregating on a 1:1 fashion. QTL mapping has been conducted through single-marker, Interval Mapping (IM) (Lander and Botstein 1989) or Composite Interval Mapping (CIM) analyses (Zeng 1993, 1994), with methodologies devised for backcross progenies, and only for single trait–environment combinations (Jordan et al. 2004; Wei et al. 2006; Raboin et al. 2008; Pinto et al. 2010; Costet et al. 2012; Nibouche et al. 2012; Singh et al. 2013). A few exceptions exist, with some studies making use of markers with a 3:1 segregation pattern or higher dosages (Aitken et al. 2008; Piperidis et al. 2008). To our knowledge, Pastina et al. (2012) described the most realistic model for QTL mapping in sugarcane to date, based on mixed models for multi-environment data. However, these authors did not include multitrait data.

In this paper, we present a QTL mapping analysis of a sugarcane segregating progeny, evaluated over multiple locations and years. Our goals were to appropriately model the MTME structure of the observations, to extend the multi-trait MIM model, in a mixed model context, to outcrossing species, i.e., progenies derived from non-inbred heterozygous parents, and to study the genetic architecture of important agricultural traits related to bioethanol and sucrose production.

## Materials and methods

### Plant material and genetic linkage map

The genetic markers utilized in this work have been previously described by Garcia et al. (2006) and Oliveira et al. (2007), and the field trial data analyzed herein have been previously addressed by Pastina et al. (2012). Briefly, we evaluated a progeny of 100 individuals obtained by a cross between Brazilian pre-commercial cultivars SP80-180 and SP80-4966 in field trials conducted in two locations (Piracicaba and Jaú, State of São Paulo, Brazil), for three consecutive harvest years (2003 through 2005). Measured traits included cane yield in Mg ${\rm ha}^{-1}$ (tonnes of cane per hectare, or TCH), sugar yield in Mg ${\rm ha}^{-1}$ (tonnes of sugar per hectare, or TSH), percent sucrose content (POL) and percent fiber content. We removed trait TSH from the analysis because of its extremely high correlation with TCH, which caused numerical problems during model fitting (data not shown). The experimental design consisted of an augmented randomized complete block design with two replicates. We separated genotypes into three groups and included four commercial checks in each of them.

These data naturally lend themselves to an MTME-based analysis because we evaluated multiple traits in multiple environments (site × harvest combinations), thus resulting in genetic and environmental correlations between traits and between environments. To have models based on more realistic assumptions, all these correlations need to be considered.

Genotypic data were available and consisted of restriction fragment length polymorphism and simple sequence repeat single-dose markers (SDMs, i.e., markers present in at most one copy) coded as dominant markers, such that only markers with 1:1 and 3:1 segregation patterns were present (Wu et al. 1992). The first situation arises when the SDM is present in a single parent, and the latter exists when the marker band is present in both parents. We applied a Chi-square test to each marker to test for segregation distortion and discarded strongly deviating markers after Bonferroni correction at a genomewise error rate of 0.05. A multipoint linkage map had previously been obtained for this population through OneMap (Wu et al. 2002; Margarido et al. 2007), with a total of 317 markers distributed over 96 linkage groups (LGs), jointly covering 2468.14 centiMorgans (cM) with the Kosambi mapping function (Kosambi 1944). This map is presented as supplementary material in Pastina et al. (2012). The number of markers per LG varied between 2 and 14. Based on shared loci, 91 out of the 96 LGs had been assembled into 11 putative homology groups (HGs), each containing from 2 to 23 LGs (Pastina et al. 2012). The other 424 markers did not map to any linkage group, and we included them in the analysis as single markers.

For notation purposes, following the convention from Wu et al. (2002), we denoted SDM loci segregating exclusively for parent SP80-180 (SP80-4966) by type $D_1 (D_2)$ and markers informative for both parents by type *C*. Note that some LGs had a mixture of all marker types, whereas others were composed solely of $D_1$ (or $D_2$) markers.

### QTL model for noninbred populations

**Fig. 1:**
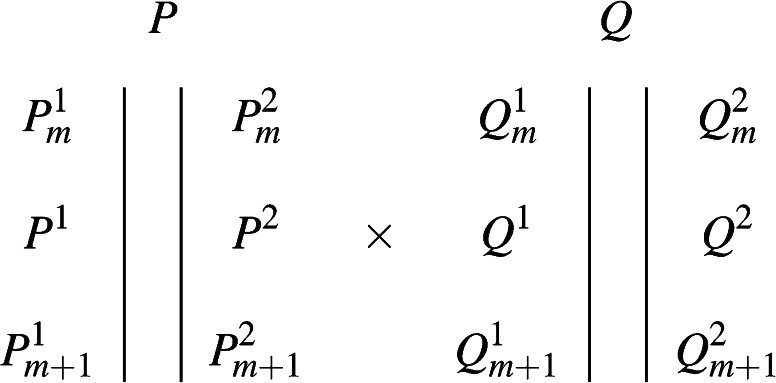
Biparental cross between non-inbred individuals *P* and *Q*. $P^{\{1,2\}}_ m,\;Q^{\{1,2\}}_m,\;P^{\{1,2\}}_{m+1}$ and $Q^{\{1,2\}}_{m+1}$ are marker alleles for loci *m* and $m+1$; $P^{1}, P^{2}, Q^{1}$ and $Q^{2}$ are QTL alleles

In a similar manner to Pastina et al. (2012) and Gazaffi et al. (2014), we consider two diploid non-inbred individuals, denoted as *P* and *Q*. For a genetic marker *m*, the two alleles of individual *P* can be denoted as $P^{1}_m$ and $P_m^{2}$, with a similar definition for both alleles of individual *Q*. Figure 1 shows a cross between two such individuals, for two adjacent markers *m* and $m+1$, and an intervening QTL with alleles $P^{1}$ & $P^{2}$ and $Q^{1}$ & $Q^{2}$ (Lin et al. 2003). Despite sugarcane being a polyploid species, in practice, we only consider two alleles because the molecular markers we used are all presence/absence dominant markers.

At any given marker or QTL locus, a diploid organism can hold up to four different alleles, which allows for a maximum of three orthogonal contrasts to be defined. For QTL alleles $P^{1}$ & $P^{2}$ and $Q^{1}$ & $Q^{2}$, the following three orthogonal contrasts have a clear genetic interpretation:

$$\begin{aligned} c_{P}= & + P^1Q^1 + P^1Q^2 - P^2Q^1 - P^2Q^2 \\ c_{Q}= & + P^1Q^1 - P^1Q^2 + P^2Q^1 - P^2Q^2 \\ c_{PQ}= & + P^1Q^1 - P^1Q^2 - P^2Q^1 + P^2Q^2 \end{aligned}$$

The first contrast compares $P^{1}$ and $P^{2}$, *i.e.*, it corresponds to the additive effect between both alleles from individual *P*. Similarly, the second contrast refers to the additive effect between alleles from individual *Q*. Finally, the third contrast tests deviations from additivity, or dominance.

Given a linkage map and a segregating progeny, conditional QTL genotype probabilities can be estimated and a mixture model devised to fit these contrasts to phenotypic data, allowing the estimation and testing of additive and dominant effects (Lander and Botstein 1989; Zeng et al. 1999). This method, however, is computationally expensive and can make generalizations to multiple QTLs and MTME data impracticable.

A commonly used approximation, initially proposed by Haley and Knott (1992), defines a linear regression model with the mathematical expectations of the effects as covariates, which makes model fitting possible via the usual least-squares estimator, available as part of many statistical packages. Both theoretical and empirical works show that such approximation generally performs almost as well as the exact model (Broman 2001). In the mixed model context, these mathematical expectations are named genetic predictors.

In the eventual scenario where a linkage group does not contain sufficient marker information for the four QTL genotypes to be identifiable (e.g., having only $D_1$-type markers), collinearity issues arise between the three contrasts such that only one or two of them can be fitted, depending on the situation (Belsley et al. 1980). As an example, consider the case where a linkage group presents markers exclusively of type $D_{1}$: This prevents recombination on individual *Q* to be identified, so only the additive effect between $P^{1}$ and $P^{2}$ can be estimated. Our model took these into consideration by only fitting effects without collinearity.

### Phenotypic model fitting

According to the experimental design implemented in the field, we fitted the following model to the phenotypic data:

$$\underline{y}_{istjkr} = \mu _{stjkr} + \underline{G}_{istjk} + \underline{\varepsilon }_{istjkr}$$

where $\underline{y}_{istjkr}$ is the phenotype of the $r{\rm th}$ replicate (block) of $i{\rm th}$ genotype in group *s*, for trait *t* in site *j* and harvest *k*; $\mu _{stjkr}$ is the mean of block *r* within group *s*, for trait *t* in site *j* and harvest *k*; $\underline{G}_{istjk}$ is the genetic effect of genotype *i* of group *s*, for trait *t* in site *j* and harvest *k*; and $\underline{\varepsilon }_{istjkr}$ is the non-genetic effect. Underlined terms represent random effects. This model will henceforth be called “genetic effects model”.

According to their origin, we separated genotypes into two groups, $n = n_g + n_c$, where $n_g$ is the number of genotypes in the progeny (clones) ($i = 1, \ldots , n_g$) and $n_c$ is the number of checks ($i = n_g+1, \ldots , n_g+n_c$). The model for $\underline{G}_{istjk}$ is given by:

$$\underline{G}_{istjk} = \left\{ \begin{array} {lll} \underline{g}_{ite} & \quad i=1, \ldots , n_g\\ c_{istjk} & \quad i=n_g+1, \ldots , n_g+n_c \end{array} \right.$$

where $\underline{g}_{ite}$ is a random variable for the genetic effect of genotype *i* for trait *t* in environment *e* and $c_{istjk}$ represents a fixed effect for check *i* in group *s* for trait *t* in site *j* and harvest *k*. Note that, for the random effect $\underline{g}_{ite}$, we combined site and harvest into an environment variable due to software limitations.

Main fixed effects of blocks, groups, traits, sites and harvests, as well as their possible interactions, were not of direct interest; thus, they were all included in the term $\mu _{stjkr}$ rather being modeled individually, simply to control for their presence. Consequently, we could obtain unbiased estimates for the effects of interest, particularly $\underline{G}_{istjk}$ (Verbyla et al. 2003; Boer et al. 2007).

The vector of genetic effects ${{\underline{\varvec{{g}}}}} = (g_{1111}, \ldots , g_{ITJK})$ follows a multivariate normal distribution, ${{\underline{\varvec{{g}}}}} \sim N(\mathbf 0 , {\varvec{G}} \otimes {\varvec{I}}_{n_{g}})$, where $\otimes$ represents the Kronecker (direct) product between two matrices and ${\varvec{I}}_{n_{g}}$ is an identity matrix of size $n_{g}$. Note that the effect of group *s* is not included in this random variable. As a first step in the model fitting process, we examined several different structures for matrix ${\varvec{G}}$ (Table 1). Two main classes of models can be distinguished: models 1 through 4 combine traits and environments factorially into different “traits”, in a broader sense of the term, and fit a single matrix ${\varvec{G}}$ to these new “traits”. Models 5 through 10, on the other hand, fit two individual component matrices, denoted ${\varvec{G}}^{{\rm trait}}$ and ${\varvec{G}}^{{\rm env}}$, respectively, for traits and environments. For the latter models, it follows that ${\varvec{G}} = {\varvec{G}}^{{\rm trait}} \otimes {\varvec{G}}^{{\rm env}}$ (Smith et al. 2007). Due to software limitations, we could decompose the (co)variance matrix into only two component matrices, which required the combination of sites and harvests into environments and forcefully prevented a more refined model to be fitted. This and all subsequent steps were performed in Genstat version 16 (VSN International 2013).

In Table 1, model 1 corresponds to a diagonal matrix, i.e., a model in which a different genetic variance is assigned to each “trait”, with all correlations being equal to zero. Model 2 also fits a different variance for each class, but includes a uniform genetic correlation. The first-order factor analytic model 3 is a multiplicative model that allows heterogeneity in both variances and covariances, i.e., approximates a fully unstructured model, while using a smaller number of parameters (Piepho 2000; Eeuwijk et al. 2001). Finally, the unstructured model 4 fits an individual (co)variance term for each trait–environment combination. Models 5 through 10 use these same structures, separately for each component matrix, in several distinct combinations. Note that all these models allow heterogeneous variances for the various traits. Additionally, the genetic effects model includes a different mean for each trait. This modeling strategy takes into account the fact that phenotypic traits are measured in different scales (i.e., data do not need to be standardized). We compared all models based on their AIC (Akaike Information Criterion) and BIC (Bayesian Information Criterion) values, where a smaller value corresponds to a better model (Burnham and Anderson 2004). Notably, we also evaluated other component matrix combinations (such as a ${\rm DIAG}\otimes {\rm DIAG}$, for example), but these resulted in poor fits to the data and are thus not shown.

**Table 1:** Genetic (co)variance matrix (${\varvec{G}}$): evaluated models

${\varvec{G}}$ matrix	Model type	$\# \ {\rm PAR}^a$	Description
${\varvec{G}} = {\varvec{G}}_{M \times M}^{{\rm trait-env}}$	1) DIAG	*M*	Heterogeneous genetic variances
	2) $\hbox {CS}_{Het}$	$M+1$	Compound symmetry (uniform correlation) and heterogeneous variances
	3) FA1	2*M*	First-order factor analytic
	4) US	$\frac{M(M+1)}{2}$	Unstructured
${\varvec{G}} = {\varvec{G}}_{T \times T}^{{\rm trait}} \otimes {\varvec{G}}_{E \times E}^{{\rm env}}$	5) DIAG $\otimes$ FA1	$\left( T + 2E\right) - 1$	Heterogeneous variation for traits and first-order factor analytic model for environments
	6) $\hbox {CS}_{Het}$ $\otimes$ FA1	$\left( T + 1 + 2E\right) - 1$	Heterogeneous compound symmetry for traits and first-order factor analytic model for environments
	7) US $\otimes$ FA1	$\left[ \frac{T\left( T+1\right) }{2} + 2E\right] - 1$	Unstructured model for traits and first-order factor analytic for environments
	8) DIAG $\otimes$ US	$\left[ T + \frac{E\left( E+1\right) }{2}\right] - 1$	Heterogeneous variation for traits and unstructured model for environments
	9) $\hbox {CS}_{Het}$ $\otimes$ US	$\left[ T + 1 + \frac{E\left( E+1\right) }{2}\right] - 1$	Heterogeneous compound symmetry for traits and unstructured model for environments
	10) US $\otimes$ US	$\left[ \frac{T\left( T+1\right) + E\left( E+1\right) }{2}\right] - 1$	Unstructured model for both traits and environments

Models 1 through 4 use the factorial combination of traits and environments as different “traits”. Models 5 through 10 use the direct product between two component (co)variance matrices for traits and environments. ^a^ Number of parameters for models 5 through 10 corresponds to the sum of parameters for each matrix, minus one necessary constraint to ensure identifiability. *M* = *T* × *E*, where *T* is the number of traits and *E* is the number of environments; *E* = *J* × *K*, where *J* is the number of sites and *K* the number of harvests. Adapted from Pastina et al. (2012) to include multiple traits

Taking into consideration the fact that the three studied variables are measured in different scales and hence have different orders of magnitude for their corresponding (co)variances, we also examined adequate models for the non-genetic residual error term $\underline{\varepsilon }_{istjkr}$. We accomplished this by testing several (co)variance structures for its associated matrix ${\varvec{R}}$, in a manner similar to that described for ${\varvec{G}}$. In particular, we evaluated models with a single matrix for factorial combinations of traits, sites and harvests, as well as models with three component matrices (trait $\otimes$ site $\otimes$ harvest), which was in this case allowed by the software. In the first group of models, we compared the fit of a diagonal model with different variances, compound symmetry with heterogeneous variances, first-order factor analytic and the fully unstructured model. In the latter group, we compared twenty-four distinct combinations of appropriate component matrices, in this case including an auto-regressive model for harvest years. We employed the same criteria for model comparison as for the ${\varvec{G}}$ matrix, namely AIC and BIC.

### QTL model

After selecting the best-fit model for the experimental design, we included genotypic information for the QTL searching process. Adapting the multiple interval mapping (MIM) methodology described by Kao and Zeng (1997) and Kao et al. (1999) to a mixed model framework, i.e., using a least-squares approximation rather than the usual mixture model approach, the QTL mapping model is expressed by:

$$\begin{aligned} \underline{y}_{istjkr}= & \,\mu _{stjkr} + \sum _{w=1}^{m} \left( x_{p_{iw}} \alpha _{p_{tjkw}} + x_{q_{iw}} \alpha _{q_{tjkw}} + x_{pq_{iw}} \delta _{pq_{tjkw}} \right) \\&+ \underline{G}^{*}_{istjk} + \underline{\varepsilon }_{istjkr} \end{aligned}$$

where $\alpha _{p_{tjkw}}, \alpha _{q_{tjkw}}$ and $\delta _{pq_{tjkw}}$ are the additive genetic predictor effects for parents *P* and *Q* and the dominance genetic predictor effect, respectively, specific for each trait × site × harvest combination, for the QTL in genomic position *w*. The term $\underline{G}^{*}_{istjk}$ refers to the residual genetic variation, not explained by QTL, thus marked with an asterisk to be differentiated from the genetic term previously described for the genetics effect model. The (co)variance matrix used for $\underline{G}^{*}_{istjk}$ was the one selected for the model without genotypic data. Henceforth, we refer to the MIM strategy more broadly as a model searching scheme.

The significance of (fixed) QTL effects was tested through the Wald test, with the null hypothesis $H_0$ defined by:

$$H_0:\left\{ \begin{array} {lllllllll} \alpha _{p_{111w}}&=& \alpha _{p_{112w}} & = & \ldots & = & \alpha _{p_{TJKw}}&=& 0 \\ \alpha _{q_{111w}}&=& \alpha _{q_{112w}} & = & \ldots & = & \alpha _{q_{TJKw}}&=& 0 \\ \delta _{{pq}_{111w}}&=& \delta _{{pq}_{112w}} & = & \ldots & = & \delta _{{pq}_{TJKw}}&=& 0 \end{array} \right.$$

The above hypothesis tests for the presence of at least one effect different from zero, i.e., if the locus at hand affects the expression of at least one trait, in at least one site and harvest combination.

#### QTL search

As originally proposed, the MIM methodology allows for several model searching strategies (Kao and Zeng 1997; Kao et al. 1999; Zeng et al. 1999). In the present study, we opted for sequential forward searches, with intervening significance checks and refining steps.

In detail, starting from the genetic effects model, we sequentially conducted one-dimensional searches for QTL and kept positions with significant effects in the model. During these searches, we jointly tested for QTL and QTL × E interaction effects and later tested for QTL × E alone (see section on “Refining steps”). To correct for multiple testing, we employed a *p* value cutoff of 0.001. We initially conducted searches for pseudo-markers positioned every 1 cM on all linkage groups and subsequently for single markers. For each genomic position, we calculated the condition indexes of the genetic predictors matrix and removed non-informative contrasts, i.e., contrasts for which the condition index was greater than an empirically chosen threshold of 3.5 (Belsley et al. 1980).

We iteratively repeated the above scheme through searches for linkage groups and unlinked markers until no more significant effects could be detected. Inclusion of each new effect in the model explains away part of the phenotypic variance, thus decreasing residual variation and increasing statistical power for detecting QTL effects (Zeng 1993, 1994).

#### Refining steps

Having performed the aforementioned forward searches, we turned to some final fine-tuning steps. The first consisted of dropping one term from the model at a time and discarding effects with *p* value >0.05. This is important because the QTL found at the initial search rounds may no longer be significant after the inclusion of other QTLs in the model.

Next, we tested for QTL × site, QTL × harvest and QTL × site × harvest interactions, individually for each QTL. We excluded non-significant interaction terms from the model, provided that there were no significant higher-order interaction effects involving the term at hand.

Following the MIM strategy, QTL positions were refined by first constructing a *p* value profile for each QTL, followed by choosing the most likely position, i.e., the one resulting in the smallest *p* value. We conducted this step iteratively until no further QTL had its position altered.

It is not formally possible to test for the competing hypotheses of pleiotropy *versus* linkage in the mixed model context when QTLs are modeled as fixed effects, for two reasons. First, because the two models are not nested, a *p* value evaluation is not feasible. Second, the AIC and BIC criteria should only be used to compare models with different random terms or structures, with a common fixed part, which is not the case for the current scenario. Hence, we took an *ad hoc* approach to removing any given QTL from the model and adding an effect for each trait separately, for a window of adjacent positions. We then compared the profiles for each trait, checking whether peaks were found at the same genomic position.

Finally, we used the final model to estimate QTL effects for each trait, in each site and harvest year. We then used the corresponding standard deviations to test the significance of each effect. Individual effects were deemed significant when $\left| {\rm effect}\right| \ge 2 \times {\rm standard \ \ deviation}$, as proposed by Malosetti et al. (2008).

## Results

### Genetic effects model

Comparison of the examined (co)variance structures for matrix ${\varvec{G}}$ made it evident that models with an unstructured matrix for environments resulted in better fits, in general, according to the BIC criterion (models 8 through 10 in Table 2). The AIC selection criterion suggested that the best model for ${\varvec{G}}$ was the fully unstructured model for trait–environment combinations (model 4), which contained a total of 171 parameters. On the other hand, BIC selected the model combining an unstructured matrix ${\varvec{G}}^{{\rm trait}}$ for traits and a separate unstructured matrix ${\varvec{G}}^{{\rm env}}$ for environments (model 10). The latter model seems to be a good balance between parsimony and goodness of fit, as it allows for a fairly complex (co)variance structure without requiring a great number of parameters. Indeed, because for this dataset BIC imposes a heavier penalty on the number of parameters, simpler models are expected. Given that our ultimate goal was to map QTLs, we decided to use the model selected by BIC because a simpler model results in smaller runtime for fitting and is thus more amenable to QTL mapping, which is particularly important in the MIM context.

**Table 2:** Models for the genetic (co)variance matrix (*M* = *T* × *E*, where *T* = 3 is the number of traits and *E* = 6 is the number of environments) and corresponding AIC and BIC values

${\varvec{G}}$ matrix	Model	$\# \ {\rm PAR}$	AIC	BIC
${\varvec{G}} = {\varvec{G}}_{M \times M}^{{\rm trait-env}}$	1) DIAG	18	10129.24	10223.02
	2) $\hbox {CS}_{Het}$	19	9956.91	10053.30
	3) FA1	36	9158.88	9299.56
	4) US	171	**7867.76**	8360.13
${\varvec{G}} = {\varvec{G}}_{T \times T}^{{\rm trait}} \otimes {\varvec{G}}_{E \times E}^{{\rm env}}$	5) DIAG $\otimes$ FA1	$(3+12)-1=14$	8244.70	8328.06
	6) $\hbox {CS}_{Het}$ $\otimes$ FA1	$(4+12)-1=15$	8238.37	8324.34
	7) US $\otimes$ FA1	$(6+12)-1=17$	8218.84	8310.02
	8) DIAG $\otimes$ US	$(3+21)-1=23$	8086.46	8193.27
	9) $\hbox {CS}_{Het}$ $\otimes$ US	$(4+21)-1=24$	8082.51	8191.92
	10) US $\otimes$ US	$(6+21)-1=26$	8064.89	**8179.51**

${\varvec{G}}$ genetic (co)variance matrix; *DIAG* diagonal; $\hbox {CS}_{Het}$ heterogeneous compound symmetry; *FA1* first-order factor analytic; and *US* unstructured. Smallest AIC and BIC values are highlighted in bold font

When we tested various different (co)variance structures for the ${\varvec{R}}$ matrix, the best model was a first-order factor analytic for trait–site–harvest combinations, according to the BIC (data not shown). However, this model was excessively slow to fit, such that performing several rounds of QTL searching proved to be infeasible. Hence, for all further analyses, we used a diagonal model, with 18 different variances (one for each trait × site × harvest combination) and no covariances. For comparison, we ran a few initial QTL searches with both models and obtained vastly similar results, showing that the simpler model did not have a detrimental effect on QTL mapping.

### QTL mapping

**Fig. 2:**
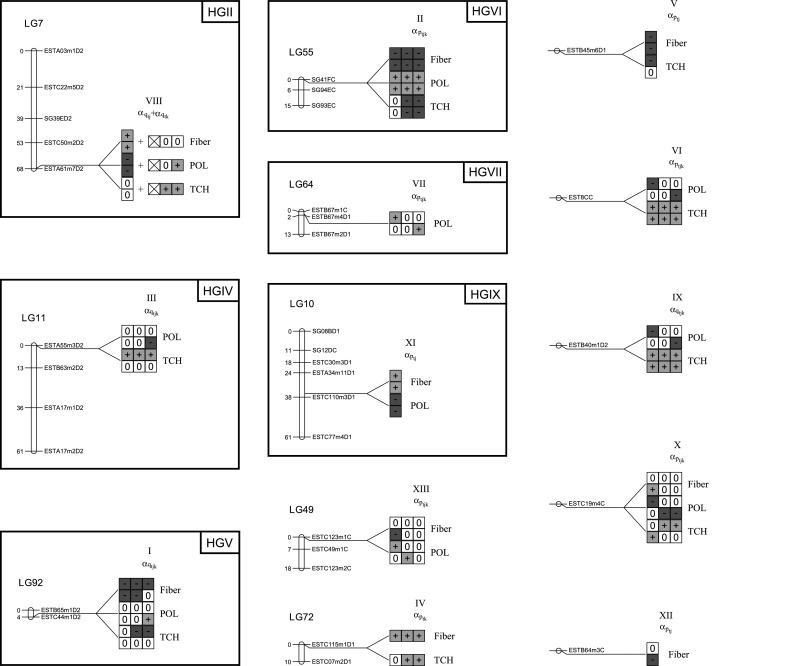
Linkage groups with detected QTL and significant effects according to the criterion $\left| {\rm effect}\right| \ge 2 \times {\rm standard \ \ deviation}$. Two lines and/or three effect columns for each trait indicate distinct effects across sites and/or harvests, respectively. Significant effects are indicated by a *plus* or *minus* sign, in case the presence of the allele increases or decreases trait expression, respectively (*Fiber* fiber content in %; *POL* sugar content; *TCH* tonnes of cane per hectare. Distances in cM using the Kosambi mapping function)

All detected QTLs are summarized in Fig. 2. Significant effects are displayed separately for each trait, site and harvest, with positive effects highlighted in green, negative effects in red and non-significant effects represented by the symbol 0. For each trait, the presence of two lines indicates that effects are different for both locations, and three columns likewise indicate different effects across harvest years. A single line (column) represents a QTL that does not interact with sites (harvests). Note that QTL numbering follows the order in which they were detected throughout the analysis, not the position along the genome as informed by the numbering of linkage groups.

We detected a total of 13 QTLs. Out of these 13 significant positions, eight were detected on a linkage group, while five were found on single, unmapped markers (Online Resource 1).

Interestingly, all QTLs interacted with sites and/or harvest years; hence, no effects were consistent across all environmental conditions evaluated (Online Resource 1). From the total of 13 terms, three interacted only with sites, specifically QTL V (marker ESTB45m6D1), XI (LG10) and XII (marker ESTB64m3C). On the other hand, QTL IV (LG72) exhibited only QTL × harvest interaction. QTL VIII (LG7) interacted both with sites and with harvests, but the three-way interaction was not significant. Finally, the remaining eight effects showed a significant three-way interaction, i.e., displayed an oscillating effect across site × harvest combinations (Online Resource 1). These results also make it apparent that very few detected effects were expressed in a more stable manner, as we observed many effects for only a single site and harvest. Nonetheless, it is interesting to observe that we did not observe any crossover interaction, as the effects for any given trait, when significant, were always consistently positive or negative across sites and harvests.

It is also important to emphasize that we determined individual significances according to the criterion $\left| {\rm effect}\right| \ge 2 \times {\rm standard \ \ deviation}$, as indicated by a colored background in Fig. 2, while we jointly tested interactions with environments through the Wald test, by dropping the appropriate effect from the model and checking the *p* value, as represented by the presence or absence of multiple effect lines or columns in those figures. This is the reason why Fig. 2 only displays effects for some of the traits, indicating that effects for the other traits were not individually significant.

Figure 2 also shows that most QTLs had pleiotropic effects, as eleven of the 13 QTLs simultaneously affected at least two traits. Of even more interest is the observation that fiber and TCH were always affected in the same direction, while sugar content was influenced in the opposite direction. Although the expression pattern of each QTL was dissimilar from the others with regard to expression in various environments, they affected the three traits in directions that agreed with phenotypic correlations, i.e., a moderate positive correlation of 0.3907 ($p < 0.0001$) between TCH and fiber and almost no correlation between sugar content and the other variables (correlation of 0.0659 between POL and TCH, with $p = 0.0232$, and a correlation of $-0.0053$ between POL and fiber, with $p = 0.8553$).

**Fig. 3:**
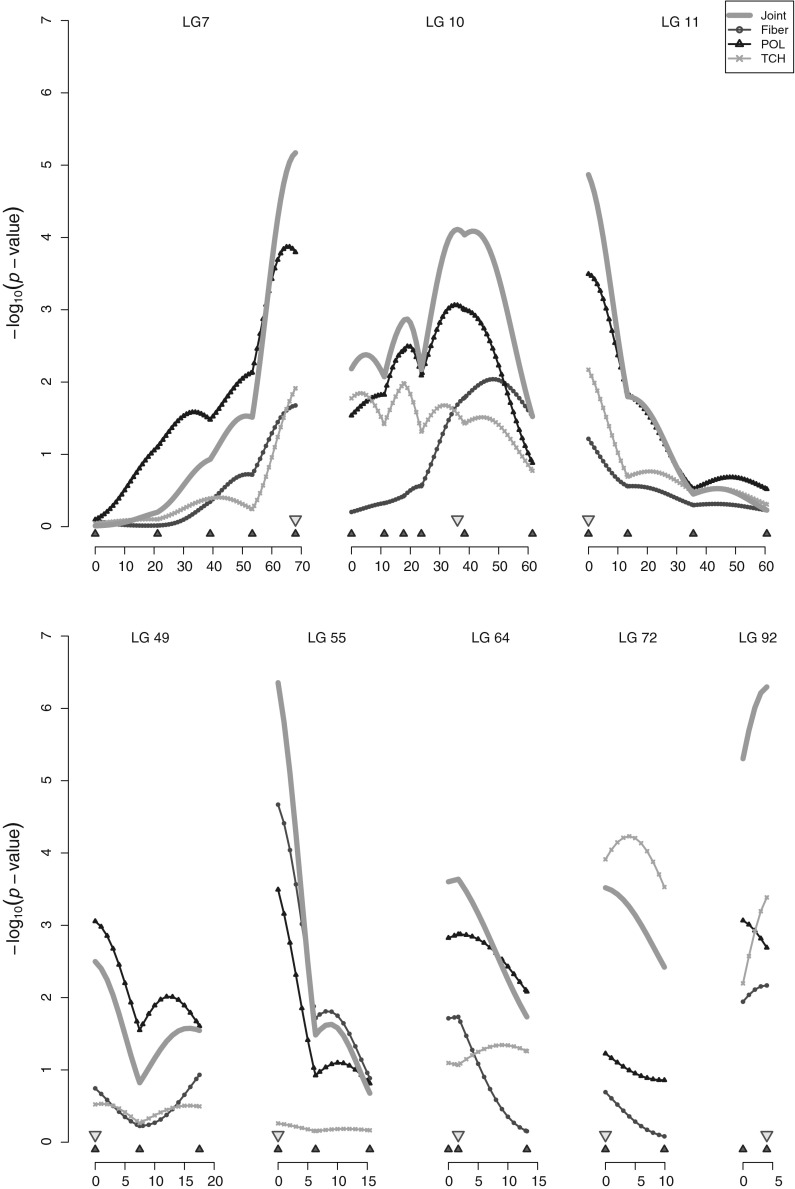
Multiple interval mapping (MIM) results indicating QTL positions (*down-pointing triangles*) and the $-{\rm log}_{10}(p{\rm -value})$ profiles along linkage groups (LG) for the joint analysis of the three traits and for each trait individually (*Fiber* percent of fiber; *POL* sugar content; *TCH* tonnes of cane per hectare. *Up-pointing triangles* molecular marker positions on linkage map. Distances in cM using the Kosambi mapping function)

For QTLs detected on linkage groups, profiles of the $-{\rm log}_{10}(p{\rm -value})$ statistic are shown in Fig. 3, both for the joint analysis of the three traits and for separate analysis of each individual trait. Visual inspection of these profiles did not provide evidence in favor of the linked QTL hypothesis over pleiotropy, for any of the detected QTLs. For some QTLs, e.g., QTL XI on LG10, peaks for all traits were found to be very close to each other. For others, such as QTL III on LG49, a significant peak was observed exclusively for one of the traits. Finally, as observed for QTL I on LG92, the linkage group was too small to allow a clear distinction of peaks.

## Discussion

Breeding programs typically leverage data collected for many traits, in multiple locations and along several years. Consequently, genetic and residual (co)variances are expected to be different across traits and environments, which in turn makes this type of data particularly suited for mixed model analysis. Proper modeling of such features decreases type I error probability, increasing the reliability of results (Piepho 2005). In this work, the selected genetic effects model explored the product of two unstructured matrices, separately for traits and environments.

Malosetti et al. (2008), working with a maize MTME dataset, reported that using a direct product of component matrices resulted in a good fit to the data, while considerably reducing the number of parameters. On the other hand, Malosetti et al. (2006, 2008) argue that unstructured models can be problematic when the number of traits or environments gets moderately large, due to numerical issues when fitting the model. Notwithstanding, the number of parameters to be estimated was relatively small for a study of this magnitude, with three traits and six environments, such that QTL searches were feasible even with these more complex models. This strategy reduced the number of parameters from 171 to 26 (Table 2), improving the fit of the genetic effects model and facilitating subsequent steps. Eeuwijk et al. (2001) state that unstructured models can be adequate when there are differences in the gene pools responsible for performance at each environment.

Deciding whether each effect in a model should be fixed or random depends on the nature of observations, objectives of the work and even preferences of each researcher (Boer et al. 2007). Because the 100 genotypes herein used for QTL mapping were sampled from a segregating progeny, we had no specific interest in any of them, but rather in the estimation of genetic variance as a whole. For this reason, we treated genotypes as random effects. We also saw no particular appeal in studying the average performance of these genotypes across environments, such that we included sites and harvests as fixed effects. Literature on QTL mapping with mixed models shows that different authors take QTLs as fixed or random according to their goals (Piepho 2000; Verbyla et al. 2003; Boer et al. 2007). Molecular markers are included as random effects usually when the goal is simply to control for residual genetic variation, as done by Wang et al. (1999), or to model QTL × E interaction, as done by Piepho (2000) and Verbyla et al. (2003). Conversely, fixed genetic predictors represent positions of specific interest in the genome, whose effects are to be estimated, as done by Piepho (2000) and Boer et al. (2007), among others. We considered QTLs as fixed effects, which allowed for the estimation of specific QTL effects for each site and harvest, given that the latter were also included as fixed. Such an approach is more appropriate to study QTL × E interaction than the stability of QTL expression (Piepho 2000). Due to the absence of shrinkage of fixed effects, it is important to emphasize that QTL effect estimates are overestimated and excessively optimistic (Boer et al. 2007), not to mention the well-known overestimation of QTL effects when the progeny sample size is small (Beavis 1994).

There are two contrasting approaches to analyzing G × E interaction from multi-environment data: one-stage analysis uses individual plot data as input and fits a statistical model simultaneously to all environments, while under the two-stage scheme, separate models are initially fit to each environment, from which BLUEs (best linear unbiased estimates) are obtained for each genotype to compose a genotype × environment table of means. The second step then consists of using this table, weighted or not, to model the G × E interaction (Smith et al. 2005; Welham et al. 2010). The first approach yields maximum statistical power and eliminates biases in effect estimation, while the latter speeds up analyses and allows a much greater amount of data to be handled, at the cost of (potentially) biased results and reduced power, and hence should be seen as an approximation. The present work had 4032 available data points, a reasonably small number that enabled a single-stage analysis to be conducted.

Analysis for individual traits did not provide, in any linkage group, strong evidence in favor of the linked QTL hypothesis. Therefore, we kept positions unchanged and estimated final effects jointly for all traits. According to the $\left| {\rm effect}\right| \ge 2 \times {\rm standard \ deviation}$ criterion (Fig. 2), the 13 detected effects exhibited different pleiotropy patterns. Only four of them expressed some influence over the three traits, seven had effects over two traits, and two terms affected a single trait. Pastina et al. (2012), utilizing univariate mixed models for QTL mapping with the same data, found a significant effect for TCH on marker ESTB64m3C, which herein only influenced fiber (QTL XII). However, closer inspection of QTL effects reveals that this marker also marginally influenced TCH, particularly on the first site (city of Piracicaba) (Online Resource 1). This apparent discrepancy possibly indicates that the individual significance criterion may not adequately reflect the joint significance tested by the Wald procedure. We noticed consistency between results for QTL II on LG55, which influenced fiber in both works, and for QTL IV on linkage group LG72, which influenced TCH. Indeed, Fig. 3 shows that the most pronounced peak in this linkage group happened exactly for TCH. It is interesting to note that despite the relatively large number of QTLs found in each study, there was limited overlap between them. In reality, only the three aforementioned effects were detected in common. It is possible that the multivariate analysis misses QTLs with (moderate) effects on a single trait, while univariate mapping may fail to identify QTLs with modest effects in each of the traits. This makes it evident that the task of finding QTL is not trivial and still deserves further investigation. In particular, it is crucial to consider the particularities of the QTL mapping study at hand, such as progeny sample size, statistical model employed and search strategy, when deriving conclusions about the genetic architecture of any given trait.

In terms of the genetic correlation between traits, $84.6\,\%$ of the effects (11 out of 13) influenced at least two of the traits, that is, most QTLs were pleiotropic to some degree. Furthermore, it is remarkable that all QTL exhibited the same pattern of signs of effects. In other words, all pleiotropic QTL contributed in the direction of a positive genetic correlation between fiber and TCH, but negative between fiber and POL and between POL and TCH. Any deviations happened only for minor effects, which were not statistically significant. QTL effects followed the sign of phenotypic correlation between fiber and TCH. On the other hand, POL was phenotypically uncorrelated with fiber and only marginally correlated with TCH, which does not agree with the genetic correlations. Notwithstanding, these negative genetic correlations reflect what breeders usually observe in breeding practice, that is, sugar content is genetically negatively correlated with yield-related traits (Jackson 2005). This important information at least partially explains the correlation between these traits. If QTL contributing against the phenotypic correlation had been found, they would make it feasible for MAS to partially break the correlation, through the selection of genotypes with increased value for all traits. Evans (2002) proved theoretically that pleiotropic QTLs opposing the phenotypic correlation are more easily detected, since hypothesis testing has greater power in that situation. The fact that most QTLs herein described presented the same correlation pattern provides evidence that there really are no effects of a different nature in the evaluated progeny.

Some causes of the QTL × E interaction could be investigated. The detected QTL exhibited some type of interaction, which can have important implications for MAS. Specifically, it would not be feasible to select for markers associated with QTLs with the same effect in both sites and with unaltered effects across years, which might be linked to stably expressed genes with major effects. In fact, it would be beneficial to select specific markers for each location, but whose effects would potentially oscillate as a function of different conditions throughout the years. These results apparently contrast with those from Pastina et al. (2012), where fewer interactions with sites were detected. However, even though we found few QTLs with fairly consistent effects, it is interesting to note that we did not observe variations in the signs of effects, neither across years within a given site nor between sites for a given year. Thus, selection for any QTL would not negatively affect genotype performance in the other location. Moreover, we did observe many QTLs that showed significant interaction with sites and harvests, but with highly similar effects at the six environments for isolated traits (Online Resource 1). When this happened, however, effects for the other traits oscillated considerably, which probably caused the Wald test for interactions to be significant, as this procedure jointly tests all traits. Univariate analyses would likely flag such QTLs as not interacting with environments, for a subset of the traits, which may partly explain differences in the extent of identified QTL × E between both studies.

From a breeding perspective, these observations thus collectively indicate that there is potential for selection of QTLs with somewhat broader effects, but that most of the effort has to be focused on QTLs for specific locations. In any case, such narrow-effect QTLs would not have detrimental effects in other (similar) environments. The nature of effect inconsistencies we observed would not alter selection procedures, as we did not detect crossover interactions, but suggests that selection response could be strongly influenced by particular crop conditions, hence limiting the efficiency of MAS. Because the goal of most breeding programs is to simultaneously improve various agronomically important traits, the main advantage of employing multi-trait analyses lies in depicting the pleiotropy patterns of QTL, which point to the inability of MAS to break undesirable genetic correlations.

 Pastina et al. (2012) described the use of mixed models for QTL mapping in outcrossing progenies based on multi-environment data, but restricted their analyses to a single trait and used an interval mapping (IM) approach (Lander and Botstein 1989) to identify putative QTLs, which were then fitted in a multiple-QTL model to test hypotheses and estimate QTL effects. More recently, Gazaffi et al. (2014) proposed a fixed effects model for QTL mapping in full-sib progenies that allows the segregation pattern of each QTL to be evaluated. Nonetheless, it is based on the CIM method and does not allow the analysis of multiple traits or environments. Our model aims to provide a complete framework for the QTL analysis of MTME data from full-sib progenies, with explicit modeling of genetic and residual (co)variances, more realistic model searching strategies and also the inclusion of epistatic interactions.

A notable advantage of the MIM method is that it may include epistatic terms directly in the QTL searching process (Kao and Zeng 1997; Kao et al. 1999). In the least-squares approximation context, genetic predictors for epistatic effects can be obtained by simply multiplying the appropriate genetic predictors, as they are orthogonal. Because of the restricted progeny size available in this study, we conducted tentative, exploratory searches for epistasis between the QTLs detected in our MTME dataset. We detected eight QTL × QTL interaction terms involving nine of the 13 QTLs. Interestingly, these epistatic interactions all displayed the same patterns of pleiotropy as the detected QTL. Additionally, as observed for QTL, epistatic terms extensively interacted with sites and/or harvests, such that breeding values would have to be calculated specifically for each environment, should this information be used for selection. Podlich et al. (2004) and Cooper et al. (2005) showed that epistatic interactions between QTL and the genetic background, in combination with QTL × E interaction, can be important for MAS.

Some authors have performed searches for epistatic effects through digenic approaches and analysis of variance or regression models (Hoarau et al. 2002; Aitken et al. 2006, 2008), but the lack of residual genetic variation control usually results in high false positive rates (Wang et al. 1999). The latter authors stated that just as the inclusion of cofactors in the CIM model successfully controls the influence of residual genetic variation in QTL mapping in the absence of epistasis, the inclusion of interacting markers linked to epistatic QTL increases power, accuracy and precision of QTL mapping. Our MIM approach allows these advantages to be achieved through the inclusion of epistasis as fixed or random effects in the model.

This is the only work, to the best of our knowledge, to make use of a multivariate mixed model for pleiotropic QTL searching with joint modeling of QTL × E interaction in sugarcane. Such a methodology has as its main advantages increased statistical power and reduced rates of false positives, which, collectively, make conclusions more reliable (Piepho 2005; Malosetti et al. 2008). Single-stage analysis with direct modeling of genetic (co)variances reduces biases and makes QTL effect estimates valid for MAS (Welham et al. 2010). Moreover, fitting multiple QTLs in a single joint model allows for breeding value estimates to be obtained, which can subsequently be leveraged by breeding programs. Even so, there is substantial room for improvement of the employed methodology, and extensive future efforts are still required. First, the mapping model hereby used was developed for diploid species, for which it is not possible for alleles to be present in multiple dosages, such that our conclusions are also approximations. It is necessary to develop molecular markers that allow precise estimates of the number of alleles to be obtained. To that end, there is ongoing work aiming at high-throughput SNP genotyping of polyploid species such as sugarcane (Serang et al. 2012; Garcia et al. 2013). Next, genetic mapping methodology for polyploids must be further developed to take these markers into account. Furthermore, QTL mapping models capable of estimating quantitative effects of multiple doses must be devised. Finally, although the integration of markers segregating 1:1 and 3:1 in a single map is an advancement in comparison with the two-way pseudo-testcross, we only used SDMs to construct the linkage map and discarded markers with larger copy numbers, thus reducing genome coverage. Lower marker saturation can be noted by the small number of markers per group, the small length of linkage groups and the fact that many linkage groups could not be integrated, as a result of the exclusive presence of $D_{1}$ or $D_{2}$ markers.

Nonetheless, despite the inherent limitations of the dataset, one-stage MTME analysis in sugarcane extracted unbiased information with high power from the data. Modeling of (co)variances through mixed models dismissed the need for unrealistic assumptions. Finally, the biological foundations of MIM culminated in easily interpretable results with potential application to breeding programs, especially through the enhanced understanding of the genetic architecture of important agronomic traits, as well as the possibility of estimating breeding values directly from the QTL model. The coupling of these advantages makes this methodology genuinely appropriate for handling data of this nature.

## Electronic supplementary material

Supplementary material 1 (pdf 34 KB)
