# Supplementary material for: Multi-trait multi-environment quantitative trait loci mapping for a sugarcane commercial cross provides insights on the inheritance of important traits
Source: Mol Breed. 2015 Aug 9;35(8):175. doi: 10.1007/s11032-015-0366-6 (PMC4529881; doi:10.1007/s11032-015-0366-6)
Supplement: Supplementary file 1 — Supplementary material 1 (pdf 34 KB) [file 11032_2015_366_MOESM1_ESM.pdf]

Multi-trait multi-environment quantitative trait loci mapping for a sugarcane commercial cross provides insights on the inheritance of important traits

Molecular Breeding

G.R.A. Margarido, M.M. Pastina, A.P. Souza, A.A.F. Garcia

A.A.F. Garcia
Departamento de Genética, Escola Superior de Agricultura "Luiz de Queiroz" (ESALQ), Universidade de São Paulo (USP)
Piracicaba-SP, Brazil
augusto.garcia@usp.br

Supplementary Table 1: QTL effect estimates under the final Multiple Interval Mapping (MIM) model, with corresponding standard deviations inside parentheses (Fiber: percent of fiber; POL: sugar content; TCH: tonnes of cane per hectare; $\alpha_{p_{tjk}}$, $\alpha_{q_{tjk}}$ and $\beta_{(\tau_1\tau_2)_{tjk}}$ are the additive effects for parents P and Q and the epistatic interaction, respectively, for each trait (t), site (j) and harvest (k). Epistatic effects are defined similarly. Boldface figures are significant according to the criterion: $|\text{effect}| \geq 2 \times$ standard deviation)

QTL	LG (effects)	Markers	Position (cM)	Trait	Site	Harvest 1	Harvest 2	Harvest 3
I	92 ($\alpha_{q_{tjk}}$)	ESTC44m1D2	4.01	Fiber	1	**−0.257** (0.110)	**−0.386** (0.126)	**−0.312** (0.132)
					2	**−0.358** (0.102)	**−0.290** (0.122)	−0.244 (0.122)
				POL	1	0.078 (0.124)	0.044 (0.132)	−0.018 (0.138)
					2	0.094 (0.114)	0.186 (0.129)	**0.452** (0.149)
				TCH	1	−3.434 (1.787)	**−4.752** (2.032)	**−6.207** (2.115)
					2	−0.617 (1.660)	−1.729 (1.915)	−2.874 (1.935)
II	55 ($\alpha_{p_{tjk}}$)	SG41FC	0.00	Fiber	1	**−0.458** (0.209)	**−0.800** (0.238)	**−0.724** (0.250)
					2	**−0.892** (0.193)	**−0.614** (0.230)	**−0.691** (0.230)
				POL	1	**0.651** (0.235)	**0.505** (0.250)	**0.611** (0.261)
					2	**0.547** (0.216)	**0.916** (0.244)	**1.203** (0.283)
				TCH	1	−6.017 (3.38)	**−9.958** (3.847)	**−9.775** (4.005)
					2	−5.622 (3.141)	**−8.251** (3.625)	**−8.735** (3.661)
III	11 ($\alpha_{q_{tjk}}$)	ESTA55m3D2	0.00	Fiber	1	−0.028 (0.112)	−0.062 (0.127)	0.007 (0.134)
					2	−0.044 (0.104)	−0.200 (0.123)	−0.164 (0.124)
				POL	1	−0.179 (0.126)	0.062 (0.134)	−0.025 (0.140)
					2	0.043 (0.116)	−0.080 (0.131)	**−0.323** (0.151)
				TCH	1	**5.174** (1.814)	**6.924** (2.058)	**6.635** (2.140)
					2	3.298 (1.691)	3.402 (1.944)	2.267 (1.966)
IV	72 ($\alpha_{p_{tk}}$)	ESTC115m1D1	0.00	Fiber	1, 2	**0.258** (0.101)	**0.272** (0.115)	**0.258** (0.120)
				POL	1, 2	−0.102 (0.111)	−0.081 (0.124)	0.011 (0.132)
				TCH	1, 2	0.260 (1.629)	**4.048** (1.846)	**4.465** (1.919)
V	NM ($\alpha_{p_{tj}}$)	ESTB45m6D1		Fiber	1	**−0.262** (0.114)	**−0.262** (0.114)	**−0.262** (0.114)
					2	**−0.360** (0.106)	**−0.360** (0.106)	**−0.360** (0.106)
				POL	1	−0.102 (0.127)	−0.102 (0.127)	−0.102 (0.127)
					2	−0.062 (0.116)	−0.062 (0.116)	−0.062 (0.116)
				TCH	1	**−3.773** (1.836)	**−3.773** (1.836)	**−3.773** (1.836)
					2	−1.232 (1.708)	−1.232 (1.708)	−1.232 (1.708)

QTL	LG (effects)	Markers	Position (cM)	Trait	Site	Harvest 1	Harvest 2	Harvest 3
VI	NM ($\alpha_{p_{t\,jk}}$)	EST8CC		Fiber	1	0.107 (0.145)	0.314 (0.165)	0.308 (0.174)
					2	0.172 (0.134)	0.288 (0.160)	0.241 (0.160)
				POL	1	**−0.354** (0.163)	−0.097 (0.174)	−0.147 (0.181)
					2	0.116 (0.149)	−0.324 (0.169)	**−0.847** (0.196)
				TCH	1	**7.610** (2.339)	**9.442** (2.667)	**7.603** (2.778)
					2	**4.641** (2.176)	**6.119** (2.517)	**5.692** (2.544)
VII	64 ($\alpha_{p_{t\,jk}}$)	ESTB67m4D1	1.66	Fiber	1	−0.117 (0.107)	−0.236 (0.122)	−0.129 (0.128)
					2	−0.018 (0.099)	−0.036 (0.118)	0.024 (0.118)
				POL	1	**0.279** (0.121)	0.068 (0.128)	0.058 (0.134)
					2	0.142 (0.111)	0.164 (0.125)	**0.419** (0.145)
				TCH	1	−0.065 (1.736)	−1.478 (1.971)	−0.590 (2.050)
					2	−0.773 (1.613)	−2.039 (1.857)	−0.710 (1.875)
VIII	7 ($\alpha_{q_{t\,j}} + \alpha_{q_{t\,k}}$)	ESTA61m7D2	68.04	Fiber	1	**0.334** (0.106)	**0.334** (0.106) − 0.035 (0.051)	**0.334** (0.106) − 0.042 (0.062)
					2	**0.234** (0.097)	**0.234** (0.097) − 0.035 (0.051)	**0.234** (0.097) − 0.042 (0.062)
				POL	1	**−0.247** (0.117)	**−0.247** (0.117) + 0.089 (0.061)	**−0.247** (0.117) + **0.220** (0.075)
					2	**−0.302** (0.107)	**−0.302** (0.107) + 0.089 (0.061)	**−0.302** (0.107) + **0.220** (0.075)
				TCH	1	2.274 (1.710)	2.274 (1.710) + **2.110** (0.829)	2.274 (1.710) + **2.283** (1.001)
					2	2.621 (1.563)	2.621 (1.563) + **2.110** (0.829)	2.621 (1.563) + **2.283** (1.001)
IX	NM ($\alpha_{q_{t\,jk}}$)	ESTB40m1D2		Fiber	1	0.163 (0.119)	0.207 (0.135)	0.065 (0.142)
					2	0.127 (0.110)	0.076 (0.130)	0.089 (0.131)
				POL	1	**−0.402** (0.133)	−0.236 (0.142)	−0.062 (0.148)
					2	−0.098 (0.123)	−0.229 (0.138)	**−0.371** (0.160)
				TCH	1	**6.109** (1.921)	**8.188** (2.183)	**8.282** (2.268)
					2	**4.090** (1.786)	**6.391** (2.056)	**5.423** (2.080)

QTL	LG (effects)	Markers	Position (cM)	Trait	Site	Harvest		
						1	2	3
X	NM ($\alpha_{p_t jk}$)	ESTC19m4C		Fiber	1	0.146 (0.145)	0.300 (0.165)	0.246 (0.174)
					2	**0.302** (0.134)	0.281 (0.160)	0.193 (0.160)
				POL	1	**−0.582** (0.163)	−0.310 (0.174)	−0.196 (0.181)
					2	−0.210 (0.150)	**−0.358** (0.169)	**−0.624** (0.196)
				TCH	1	3.613 (2.345)	**6.408** (2.669)	**7.488** (2.778)
					2	**4.443** (2.180)	4.223 (2.516)	3.108 (2.541)
XI	10 ($\alpha_{p_t j}$)	ESTA34m11D1/	36.00	Fiber	1	**0.305** (0.113)	**0.305** (0.113)	**0.305** (0.113)
					2	**0.258** (0.104)	**0.258** (0.104)	**0.258** (0.104)
		ESTC110m3D1		POL	1	**−0.393** (0.125)	**−0.393** (0.125)	**−0.393** (0.125)
					2	**−0.377** (0.115)	**−0.377** (0.115)	**−0.377** (0.115)
				TCH	1	−0.357 (1.812)	−0.357 (1.812)	−0.357 (1.812)
					2	2.417 (1.685)	2.417 (1.685)	2.417 (1.685)
XII	NM ($\alpha_{p_t j}$)	ESTB64m3C		Fiber	1	−0.328 (0.188)	−0.328 (0.188)	−0.328 (0.188)
					2	**−0.350** (0.174)	**−0.350** (0.174)	**−0.350** (0.174)
				POL	1	−0.297 (0.209)	−0.297 (0.209)	−0.297 (0.209)
					2	0.111 (0.192)	0.111 (0.192)	0.111 (0.192)
				TCH	1	4.777 (3.018)	4.777 (3.018)	4.777 (3.018)
					2	−1.313 (2.813)	−1.313 (2.813)	−1.313 (2.813)
XIII	49 ($\alpha_{p_t jk}$)	ESTC123m1C	0.00	Fiber	1	−0.229 (0.221)	−0.389 (0.252)	−0.463 (0.265)
					2	**−0.441** (0.204)	−0.265 (0.244)	−0.375 (0.244)
				POL	1	**0.656** (0.248)	0.372 (0.265)	0.409 (0.276)
					2	−0.023 (0.228)	**0.640** (0.258)	0.388 (0.300)
				TCH	1	−0.826 (3.575)	−0.355 (4.074)	−0.990 (4.241)
					2	0.450 (3.321)	1.105 (3.839)	−2.886 (3.878)
